# Multiple Lineages of Human Breast Cancer Stem/Progenitor Cells Identified by Profiling with Stem Cell Markers

**DOI:** 10.1371/journal.pone.0008377

**Published:** 2009-12-21

**Authors:** Wendy W. Hwang-Verslues, Wen-Hung Kuo, Po-Hao Chang, Chi-Chun Pan, Hsing-Hui Wang, Sheng-Ta Tsai, Yung-Ming Jeng, Jin-Yu Shew, John T. Kung, Chung-Hsuan Chen, Eva Y.-H. P. Lee, King-Jen Chang, Wen-Hwa Lee

**Affiliations:** 1 Genomics Research Center, Academia Sinica, Taipei, Taiwan; 2 Department of Surgery, National Taiwan University Hospital, Taipei, Taiwan; 3 Department of Pathology, National Taiwan University Hospital, Taipei, Taiwan; 4 Institute of Molecular Biology, Academia Sinica, Taipei, Taiwan; 5 Department of Development and Cell Biology, University of California Irvine, Irvine, California, United States of America; 6 Department of Biological Chemistry, University of California Irvine, Irvine, California, United States of America; Roswell Park Cancer Institute, United States of America

## Abstract

Heterogeneity of cancer stem/progenitor cells that give rise to different forms of cancer has been well demonstrated for leukemia. However, this fundamental concept has yet to be established for solid tumors including breast cancer. In this communication, we analyzed solid tumor cancer stem cell markers in human breast cancer cell lines and primary specimens using flow cytometry. The stem/progenitor cell properties of different marker expressing-cell populations were further assessed by *in vitro* soft agar colony formation assay and the ability to form tumors in NOD/SCID mice. We found that the expression of stem cell markers varied greatly among breast cancer cell lines. In MDA-MB-231 cells, PROCR and ESA, instead of the widely used breast cancer stem cell markers CD44^+^/CD24^-/low^ and ALDH, could be used to highly enrich cancer stem/progenitor cell populations which exhibited the ability to self renew and divide asymmetrically. Furthermore, the PROCR^+^/ESA^+^ cells expressed epithelial-mesenchymal transition markers. PROCR could also be used to enrich cells with colony forming ability from MB-361 cells. Moreover, consistent with the marker profiling using cell lines, the expression of stem cell markers differed greatly among primary tumors. There was an association between metastasis status and a high prevalence of certain markers including CD44^+^/CD24^−/low^, ESA^+^, CD133^+^, CXCR4^+^ and PROCR^+^ in primary tumor cells. Taken together, these results suggest that similar to leukemia, several stem/progenitor cell-like subpopulations can exist in breast cancer.

## Introduction

The recently emerged concept of cancer stem cells has led to new hypotheses about tumor progression. Cancer stem cells can divide asymmetrically to self-renew and generate transient-amplifying tumor cells that cause tumor formation and subsequent metastasis. Thus, within the population of cancer cells, cancer stem cells are the ones which can form new tumors and their asymmetric division contributes to tumor heterogeneity. It has been reported that cancer stem cells are present in acute myelogenous leukemia (AML) [1] as well as many solid tumors [2]–[9] including breast tumors [10]. It has been demonstrated that leukemia stem cells are heterogeneous in terms of their origins [11] and different leukemia stem cells can give rise to different types of leukemia [12], [13]. However, it is not fully known whether heterogeneous cancer stem cells exist in the many types of solid tumors and how this heterogeneity may affect treatment response of these cancers.

Of the many types of breast cancers, approximately 80 percent are invasive ductal carcinomas, and 10–15 percent are invasive lobular carcinomas. Additional rare types constitute less than 5–10 percent of breast cancers. Gene expression profiling can further classify invasive ductal carcinomas into five subtypes: luminal A, luminal B, ERBB2 (human epidermal growth factor receptor 2, HER2), basal and normal-like [14]–[17]. One fundamental question that needs to be addressed is whether these different subtypes of breast cancers are derived from different lineage origins. Differing cancer stem cells in each type may explain why they differ in degree of metastasis and invasion, as well as prognosis outcome and treatment response. It is thus essential to identify and characterize these cancer stem cell populations in order to establish the origin and optimal treatment strategy of each breast cancer subtype (see [18] for review).

Breast cancer stem cells have been isolated from human breast tumors or breast cancer-derived pleural effusions using flow cytometry to find subpopulations of cells with a specific pattern of cell surface markers (CD44^+^, CD24^−/low^, ESA^+^ (epithelial specific antigen)) but lacking expression of specific lineage markers (Lin^−^) [10]. These cells expressed epithelial-mesenchymal transition (EMT) markers [19] and had higher tumorigenic potential than bulk tumor cells after transplantation in nonobese diabetic/severe combined immunodeficient (NOD/SCID) mice [10], [19]. It has also been shown that single cell suspensions of CD44^+^CD24^−/low^Lin^−^ cells from human breast cancers were able to proliferate extensively and form clonal nonadherent mammospheres in a low attachment *in vitro* culture system [20]. These mammospheres were more tumorigenic than established breast cancer-derived cell lines including MCF-7 and B3R [20].

Additional markers useful in characterizing breast cancer stem cells were recently reported [21]–[23]. PROCR, identified using gene expression profiling of primary breast cancers [22], is also a known marker of hematopoietic, neural, and embryonic stem cells [24]. An additional marker, CD133, was identified for breast cancer stem cells isolated from cell lines generated from Brca1^−exon11^/p53^+/−^ mouse mammary tumors [23] and is a known marker of cancer stem cells in several organs including brain, blood, liver and prostate [2], [3], [25], [26]. A more recent study showed that aldehyde dehydrogenase (ALDH) was increased in a subpopulation of both normal and cancerous human mammary epithelial cells that exhibit stem/progenitor cell properties. This subpopulation is tumorigenic, capable of self-renewal, and able to generate tumors that had the heterogeneity of the parental tumor [21]. Other surface markers such as CXCR4 and ABCG2 may be associated with cancer stem cell characteristics. CXCR4 is a G-coupled heptahelical receptor contributing to metastasis in breast cancers [27]. ABCG2 is one of the ABC transporters which has been detected in known stem/progenitor cells such as hematopoietic stem cells [28], nestin-positive islet-derived progenitors [29] and neural stem cells [30]. Although each of these markers has been studied individually, they have not been used together to determine the overall marker profile of individual tumors and the possible heterogeneous origins in tumors.

To address this fundamental question, we first performed cancer stem cell marker profile analysis using human breast cancer cell lines and specimens to further define and characterize different stem cell populations by flow cytometry, along with *in vitro* and *in vivo* assays to verify the stem cell properties of different cell isolates. Our results showed that: 1. the expression of stem cell markers differed greatly among breast cancer cell lines as well as primary tumors, 2. the previously recognized markers for breast cancer stem cells may not be the optimal or universal markers for identifying cancer stem cell populations, and 3. a highly tumorigenic subpopulation expressing PROCR^+^/ESA^+^ was identified. Furthermore, this subpopulation was able to divide asymmetrically both *in vitro* and *in vivo*, and expressed EMT markers. These results suggest existence of multiple subpopulations of breast cancer stem cells, as in the case for leukemia.

## Results

### Breast Cancer Cell Lines Heterogeneously Expressed Stem Cell Markers

To examine the expression profile of cancer stem cell markers in breast cancer cell lines, we selected eight known stem cell markers and performed FACS analysis in eight human breast cancer cell lines (Table 1). Among those markers, CD44 was expressed mostly in basal-like cell lines including MDA-MB-468, MDA-MB-231, and HCC1937, while CD24 was expressed in luminal-like cell lines such as T47D, MCF-7, ZR-75, and SKBR-3. ALDH expression was observed in most of the cell lines and not associated with specific cell types. Although it has been reported that HER2 overexpression could drive tumorigenesis and ALDH expression [31], our cell line data did not support such a relationship. This was consistent with the human specimen data described below which showed no strong relationship between HER2 and ALDH expression. Specifically, SKBR-3 and MDA-MB361 both expressed HER2 but differed in ALDH expression. PROCR was expressed only in mesenchymal-like MDA-MB-231 and luminal-like MDA-MB-361 cells. While ABCG2 was not expressed in all cell lines except a few MCF-7 cells, high level of CD133 was detected in almost all MDA-MB-468 cells, but not in other cell lines. Furthermore, only a small portion of ZR-75 cells, but not others, expressed CXCR4, while ESA was expressed in all the cell lines, but the level was relatively low in MDA-MB231 (Table 1). These data suggest that human breast cancer cell lines express stem cell markers heterogeneously and likely contain different types of cancer stem/progenitor cells.

**Table 1:** Different human breast cancer cell lines expressed different known solid cancer stem cell markers.

Marker/Cell line	MB468	MB231	HCC1937	T47D	MCF7	ZR75	SKBR3	MB361
ER	-	-	-	+	+	+	-	+
PR	-	-	-	+	+	-	-	-
HER2							o.e.	o.e.
CD44	+++	+++	+++	++	++	+	+	+++
CD24	+++	-	++	+++	+++	+++	+++	-
CD44^+^/CD24^-/low^	-	+++	++	-	-	-	-	+++
CD133	+++	-	-	-	-	-	-	-
PROCR	-	++	-	-/+	-	-	-	++
ABCG2	-	-	-	-	+	-	-	-
CXCR4	-	-	-	-	-	+	-	-
ESA	+++	++	+++	+++	+++	+++	+++	+++
ALDH	+	+	+	-	+	+	++	-

The ER/PR (+/−) and HER2 overexpression (o.e.) status were adapted from Neve et al (2006)[44].

−, not detectable.

+, <5%.

++, 5–70% of the cells express the marker indicated.

+++, >70% of the cells express the marker indicated.

### CD44^+^/CD24^−/low^ and ALDH Are Not the Universal Markers for Isolation of Cancer Stem Cells with High Efficiency of Colony Forming Ability

**Figure 1:**
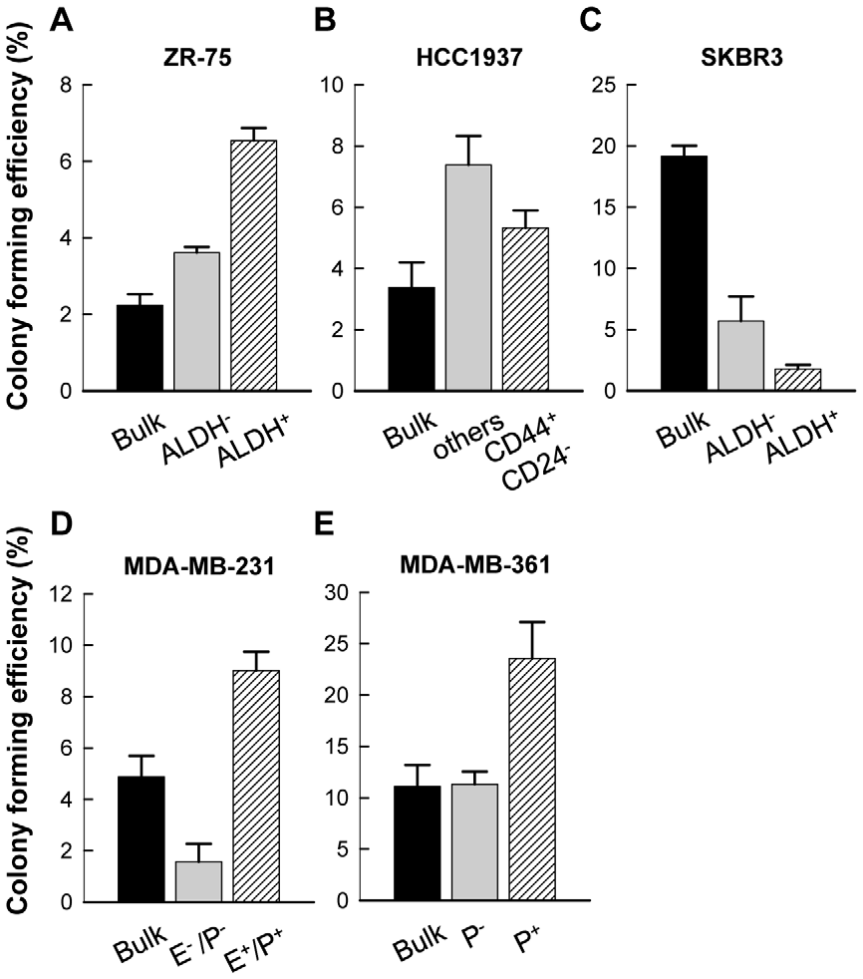
Soft agar colony forming efficiency of bulk cells or marker expressing or nonexpressing subpopulations from breast cancer cell lines. Soft agar colony formation assay of cells isolated from ZR-75 (A), HCC1937 (B), SKBR-3 (C), MDA-MB-231 (D) and MDA-MB361 (E) cells with the indicated cell surface markers expressed. The unsorted cells (Bulk) were used as control. Shown is the percentage of colony formation. Results are means ± SD of triplicate samples from one representative experiment.

The hallmark of cancer stem cells is that one or very few cells are capable of forming tumor in animal assay. To accelerate the selection procedure for identification of stem cells, we sought to use anchorage-independent growth in soft agar culture since it best mimics tumorigenic ability in animal. Although mammosphere formation has served as an *in vitro* stem cell criteria, not every breast cancer cell line posses such ability [32], [33]. Based on cell surface markers listed in Table 1, the bulk cells were labeled with antibodies specifically against the selected stem cell markers, sorted into two separate groups using fluorescence activated cell sorting (FACS), and subjected to soft agar colony formation assays (Figure 1). If the selected markers effectively identified cancer stem cells, it would be expected that the marker-expressing subpopulation would have higher colony forming efficiency than cells not expressing the markers.

Based on this premise, we have analyzed five cell lines including ZR-75, HCC1937, SKBR-3, MDA-MB-231 and MDA-MB-361. Consistent with previous reports [21], [34], ZR-75 cells sorted by ALDH^+^ were negatively associated with ER expression (Figure S1, Method S1), but exhibited higher colony forming ability than the bulk cells or ALDH^−^ cells (Figure 1A). However, if sorted by CXCR4^+^, the subpopulation exhibited less efficiency in colony forming ability when compared with the ALDH^+^ subpopulation (Figure S2, Method S1), suggesting that the ALDH^+^ subpopulation may contain more cancer stem/progenitor cells of the ZR-75 cell line. The subpopulation of HCC1937 cells sorted by CD44^+^/CD24^−/low^ formed more colonies than the bulk cells (Figure 1B, bulk vs. CD44^+^/CD24^−^). However, the remaining subpopulation, which was not CD44^+^/CD24^−/low^, also formed colonies more than the bulk cells and showed a better efficiency than the CD44^+^/CD24^−/low^ cells (Figure 1B). This suggested the existence of another stem cell population, which was not CD44^+^/CD24^−/low^. Further selection with ALDH marker from HCC1937 cells failed to enrich a subpopulation with higher colony forming ability (Figure S2, Method S1). Similarly, the subpopulation sorted by ALDH^+^ from SKBR-3 cells showed less efficiency in colony formation than the bulk and the ALDH^−^ cells (Figure 1C). Taken together, these results suggested that CD44^+^/CD24^−/low^ and ALDH may not be universal markers to identify and enrich highly tumorigenic stem cells from all breast cancers. Instead, there may be other types of breast cancer stem cells that are defined by other markers.

### PROCR^+^/ESA^+^ Enriched a Subpopulation of Breast Cancer Cells with High Colony Forming Efficiency in Soft Agar

It was noted that in MDA-MB-231 and MDA-MB-361 cell lines, almost all cells (≥90%) were CD44^+^/CD24^−/low^. However, less than five and twelve percent of the cells were able to form colonies in soft agar, respectively (Figure 1D and 1E, bulk cells). In MDA-MB-231, the sorted PROCR^+^/ESA^+^ cells showed a two-fold and nine-fold increase in colony forming efficiency when compared with that of the bulk cells and PROCR^−^/ESA^−^ cells, respectively (Figure 1D), suggesting that the PROCR^+^/ESA^+^ subpopulation may be comprised of a higher number of stem/progenitor cells than the PROCR^−^/ESA^−^ cells.

Since more than 85–90% of MDA-MB-361 cells expressed CD44 and ESA (Table 1), PROCR alone was used to evaluate whether a high tumorigenic subpopulation can be enriched in this cell line. A two-fold higher colony forming efficiency was observed in PROCR^+^ cells when compared with the bulk cells or the PROCR^−^ cells (Figure 1E). However, no difference in colony forming efficiency was observed between the bulk cells and PROCR^−^ cells, suggesting that the PROCR marker alone may not be the optimal selection of the stem/progenitor cells from this cell line.

### PROCR^+^/ESA^+^ Subpopulation Cells Were Highly Tumorigenic *In Vivo*

To test whether the selected PROCR^+^/ESA^+^ MDA-MB-231 cells form tumors, we injected 100, 500 and 2500, respectively, PROCR^+^/ESA^+^ MDA-MB-231 cells into the mammary fat pad of NOD/SCID mice. Consistent with the soft agar colony formation assays, PROCR^+^/ESA^+^ cells were highly tumorigenic since tumor formation could be observed from a starting population of as little as 100 PROCR^+^/ESA^+^ cells within 50 days (Table 2). Although PROCR^−^/ESA^−^ cells were also able to form tumors when more cells (2500 cells) were injected, the volume of the PROCR^−^/ESA^−^ derived tumors was always much less than that of PROCR^+^/ESA^+^ derived tumors. These results suggest, first, that the soft agar colony formation can serve as a surrogate assay for tumorigenicity in animals during this cancer stem cell selection procedure and; second, that the cancer stem/progenitor cells of MDA-MB-231 were enriched by PROCR^+^/ESA^+^ selection.

**Table 2:** Xenograph and tumor growth in NOD/SCID injected with PROCR^+^/ESA^+^ or PROCR^−^/ESA^−^ MDA-MB-231 cells.

Stem cell marker	Number of cells injected	Tumor formation efficiency * ^,^ † (average tumor volume, mm^3^)
PROCR^+^/ESA^+^	100	2/4, (28)
	500	3/4, (87)
	2500	3/4, (144)
PROCR^−^/ESA^−^	100	0/4, (0)
	500	0/4, (0)
	2500	1/4, (8)

* Numbers of glands that formed tumor after 50-day innoculation/Total glands injected.

† Tumor formation efficiency was measured from two sets of experiments. Tumor volume was measured in one set of the experiments.

Also consistent with the soft agar colony formation assay (Figure 1E), both PROCR^+^ and PROCR^−^ cells isolated from the MDA-MB-361 cell line were able to form tumors in the mammary fat pad of NOD/SCID mice from as little as 500 cells injected (Table 3). However, the PROCR^+^ derived tumors were five to ten-fold larger than tumors derived from PROCR^−^ cells. The observation that PROCR^−^ cells were able to cause tumor formation, albeit at a lower rate of tumor growth, suggests that the PROCR marker alone may not be the optimal selection of the stem/progenitor cells from this cell line.

**Table 3:** Xenograph and tumor growth in NOD/SCID injected with PROCR^+^ or PROCR^−^ MDA-MB-361 cells.

Stem cell marker	Number of cells injected	Tumor formation efficiency * (average tumor volume, mm^3^)
PROCR^+^	100	0/3, (0)
	500	3/3. (221)
	2500	2/3, (449)
PROCR^−^	100	0/3, (0)
	500	2/3, (22)
	2500	3/3, (87)

* Numbers of glands that formed tumor after 50-day innoculation/Total glands injected.

### PROCR^+^/ESA^+^ Subpopulation Cells Divide Asymmetrically *In Vitro* and *In Vivo*

**Figure 2:**
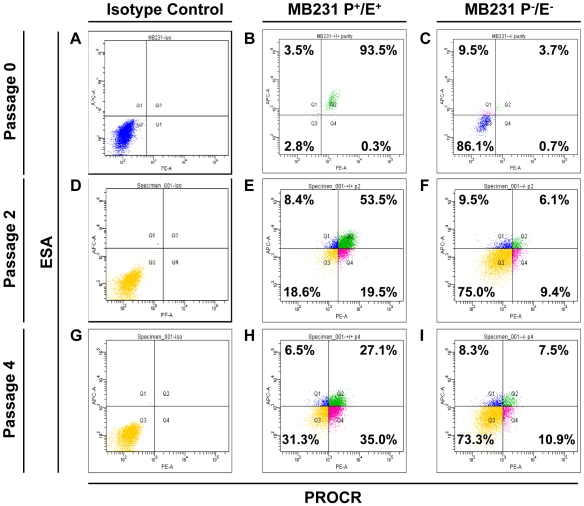
PROCR/ESA marker profiling of PROCR^+^/ESA^+^ and PROCR^−^/ESA^−^ MDA-MB-231 cells *in vitro* to evaluate their asymmetric division. PROCR^+^/ESA^+^ (B) and PROCR^−^/ESA^−^ (C) cells were sorted and cultured for four passages in an attachable culture system. The PROCR/ESA marker profile was evaluated at passage 2 (E, F) and 4 (H, I). Isotype control cells were assayed at each time point as indicated (A, D, G). The PROCR^+^/ESA^+^ subpopulation was able to asymmetrically divide into other subpopulations *in vitro*; however, the PROCR^−^/ESA^−^ subpopulation was not capable of asymmetric division.

**Figure 3:**
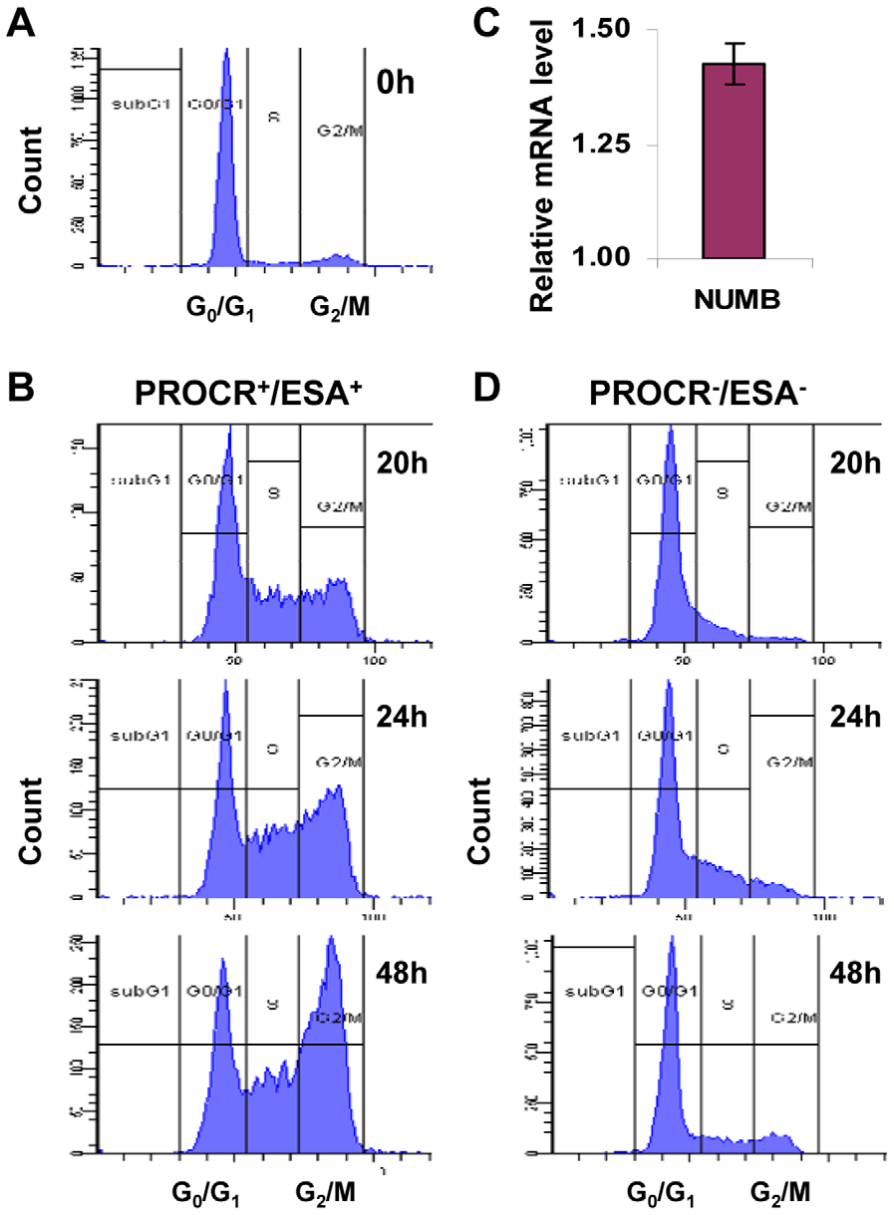
Cell cycle profiling and NUMB expression of PROCR^+^/ESA^+^ and PROCR^−^/ESA^−^ MDA-MB-231 cells. Bulk cells (A) were synchronized by serum starvation using DMEM supplemented with 0.5% FBS for 48 h. At 20, 24 and 48 h after release from serum starvation, PROCR^+^/ESA^+^ (B) and PROCR^−^/ESA^−^ (D) cells stained with propidium iodide and the cell cycle profile analyzed. C. The expression level of NUMB in PROCR^+^/ESA^+^ relative to PROCR^−^/ESA^−^ cells were assayed using real-time PCR. GAPDH was used as an internal control. Results are means ± SD of triplicate experiments.

Asymmetric division is another stem cell characteristic in addition to the high tumorigenic ability. To test whether the selected subpopulation exhibits this property, the PROCR^+^/ESA^+^ and PROCR^−^/ESA^−^ cells were sorted and cultured in an attachment culture system (Figure 2). After just two passages, the prevalence of PROCR^+^/ESA^+^ cells decreased from 93.5% (Figure 2B) to 53.5% (Figure 2E). With an additional two passages, the prevalence of this population decreased to 27.1% (Figure 2H), and the prevalence of other subpopulations increased. Thus, after only two to four passages, the proportion of each subpopulation had already returned to that of the bulk population before sorting. To further confirm whether the increased prevalence of the PROCR^−^/ESA^−^ subpopulation was indeed due to asymmetric division, cell cycle profiling analysis was performed (Figure 3). After synchronization (Figure 3A), the cells were released from serum starvation and a time-course cell cycle profiling analysis was performed for the PROCR^+^/ESA^+^ and PROCR^−^/ESA^−^ cells. The PROCR^+^/ESA^+^ cells were able to reenter the cell cycle earlier and enter G2/M phase faster (Figure 3B) than the PROCR^−^/ESA^−^ cells (Figure 3D). Also, the PROCR^+^/ESA^+^ cells expressed higher levels of NUMB which has been shown to regulate asymmetric division in several cell types [35]–[37] (Figure 3C). These observations demonstrated that the increased prevalence of the PROCR^−^/ESA^−^ cells (Figure 2E, 2H) did not result from the proliferation of PROCR^−^/ESA^−^ cells, but rather was due to the proliferation and asymmetric division of PROCR^+^/ESA^+^ cells. In contrast, the sorted PROCR^−^/ESA^−^ cells were not able to divide asymmetrically. The prevalence of PROCR^−^/ESA^−^ cells was 86% (Figure 2C) when sorted for *in vitro* culture. After 2 and 4 passages, the prevalence remained at 75% (Figure 2F) and 73% (Figure 2I), respectively. The minor changes in the prevalence of other subpopulations from passage 2 to passage 4 were negligible (Figure 2F, 2I).

To further address whether such asymmetric dividing ability also occurred *in vivo*, tumors derived from PROCR^+^/ESA^+^ MDA-MB-231 cells were excised from the NOD/SCID mice and the PROCR and ESA expression profiles examined after separation of mouse lineages from the extracted tumor cells. The results indicated a decrease in the prevalence of the PROCR^+^/ESA^+^ cells from 93.8% (before injection, Figure S3) to only 0.6% (Figure S3, Method S1), and an increase in other subpopulations. Interestingly, this low percentage *in vivo* reflected the prevalence of PROCR^+^ cells in the human breast cancer specimens tested (see below).

### PROCR^+^/ESA^+^ Subpopulation Cells Expressed EMT Markers

**Figure 4:**
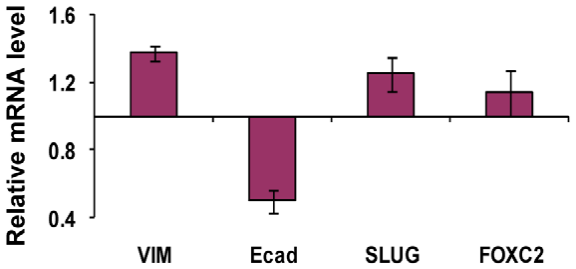
PROCR^+^/ESA^+^ cells express markers associated with epithelial-mesenchymal transition (EMT). Gene expression levels of vimentin, E-cadherin, Slug and FOXC2 in PROCR^+^/ESA^+^ relative to PROCR^−^/ESA^−^ cells were assayed using real-time PCR. GAPDH mRNA was used as an internal control. Results are means ± SD of triplicate experiments.

It has been reported that stem cells from human mammary glands and mammary carcinomas express EMT markers including increased vimentin, SLUG and FOXC2 gene expression and decreased E-cadherin expression [19]. We then tested whether the PROCR^+^/ESA^+^ subpopulation of MDA-MB-231 cells exhibited this property by real-time RT-PCR analysis of EMT-marker gene expression (Figure 4). Consistent with the other indicators of stem cell properties described above, we found that expression of vimentin, SLUG and FOXC2 was increased in PROCR^+^/ESA^+^ cells relative to PROCR^−^/ESA^−^ cells, while expression of E-cadherin was lower. Taking these results together, the PROCR^+^/ESA^+^ subpopulation of MDA-MB-231 cells was indeed comprised of cancer stem cells.

### Breast Cancer Cells Freshly Prepared from Clinical Specimens Express Different Stem Cell Markers

**Figure 5:**
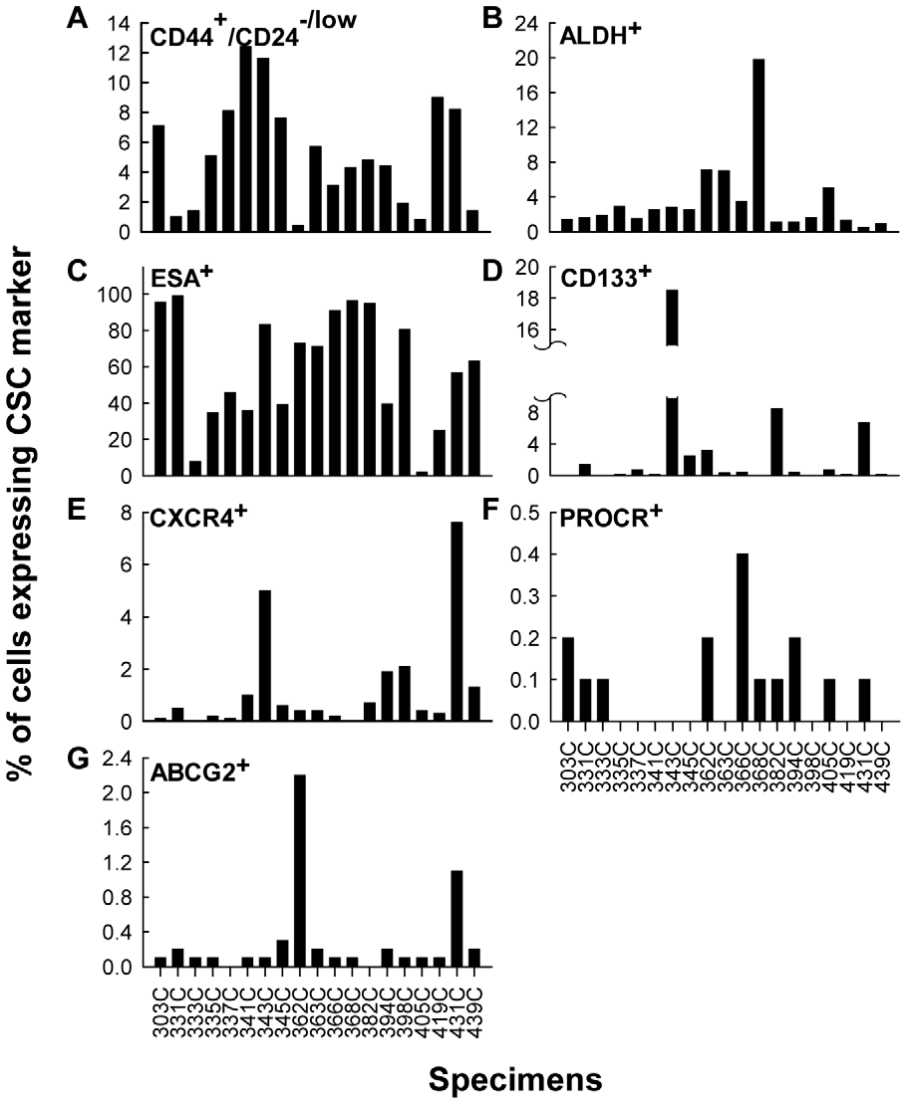
Stem cell marker profiling of primary human breast cancer specimens. Known breast cancer stem cell markers CD44^+^/CD24^−/low^ (A), ALDH (B) and ESA (C), and other solid cancer stem cell markers CD133 (D), CXCR4 (E), PROCR (F) and ABCG2 (G) were detected in primary human breast cancer specimens using flow cytometry after cell labeling. Each panel shows the percentage of cells from each primary human specimen expressing the indicated stem cell marker. Results are from one experiment since the limited material allowed only one analysis.

To investigate whether breast cancer cells freshly derived from clinical specimens express heterogeneous stem cell markers as observed in human breast cancer cell lines, we performed stem cell marker profiling by FACS analysis of nineteen specimens obtained from National Taiwan University Hospital. All specimens examined contained CD44^+^/CD24^−/low^ cells; however, the prevalence of these cells ranged from 1 to 12.4 percent (Figure 5A) and that of ALDH^+^ cells ranged from 1.4 to 19.8 percent (Figure 5B). The percentage of cells that expressed CD133, CXCR4 and ESA varied even more among specimens. Of the nineteen specimens, all specimens contained ESA^+^ cells (Figure 5C); however, only eleven specimens contained a detectable number of CD133^+^ cells (Figure 5D) and fifteen contained CXCR4^+^ cells (Figure 5E). In specimens where expression was detectable, the percentages of the cells that expressed these markers ranged from 2.1 to 99 percent, 0.1 to 18.5 percent and 0.1 to 7.6 percent for ESA, CD133 and CXCR4, respectively. PROCR and ABCG2 expressing cells were rare: only 0.1 to 0.4 percent of the cells from ten specimens had detectable PROCR expression (Figure 5F) and 0.1 to 2.2 percent of the cells from seventeen specimens had detectable ABCG2 expression (Figure 5G). It was noted that all specimens were collected prior to chemotherapy or hormone therapy. Thus, the expression of ABCG2 may not be resulted from these treatments. Consistent with the cell line data described above, these primary tumor cells expressed heterogeneous stem cell markers, suggesting the existence of different types of cancer stem/progenitor cells.

To search for any potential clinical correlation with those marker expressing profiles, pathology data of each patient was obtained from NTU Hospital (Table 4) and analyzed by statistics. Of the nineteen patients, twelve were estrogen receptor (ER) positive, nine were progesterone receptor (PR) positive, and three were HER2 overexpressed. Ten of the patients had metastasis to lymph nodes. Despite the limited sample size, the data revealed an overall trend that the cancer specimens with higher numbers of CD44^+^/CD24^−/low^ cells express low level of HER2 (Table 4, Figure 5A). However, a positive association between the prevalence of CD44^+^/CD24^−/low^ cells and ER status was not found (Table 4, Figure 5A). These results were consistent with the data obtained from breast cancer cell lines (Table 1) and similar to the previously reported [38].

**Table 4:** Pathology data of the human breast cancer specimens tested.

Specimen	Age	Size	Lymph node	ER (%)	PR (%)	HER2
303C	43	2.9	3/17 (+)	90	30–40	1 (L)
331C	84	3.5	0/21 (−)	>90	40	1 (L)
333C	51	4.5	0/11 (−)	0	0	1–2 (M)
335C	57	2.5	0/10 (−)	0	0	1 (L)
337C	42	1.8	1/4 (+)	>90	20–50	1 (L)
341C	56	3	0/25 (−)	90	<5	0 (ND)
343C	51	3.5	7/8 (+)	95	0	0 (ND)
345C	57	5	0/1 (−)	0	0	1 (L)
362C	44	NA	NA	0	0	3 (H)
363C	53	5	0/3 (−)	90	90	0 (ND)
366C	43	4.5	12/29 (+)	60	30	1 (L)
368C	47	5	3/30 (+)	90	0	0 (ND)
382C	86	5.6	6/8 (+)	>90	5–10	0 (ND)
394C	52	7.3	26/35 (+)	0	0	3 (H)
398C	65	2.5	7/24 (+)	70	0	1–3 (M)
405C	36	8	35/49 (+)	20	2	3 (H)
419C	76	3.3	0/9 (−)	0	0	0 (ND)
431C	44	2.8	2/22 (+)	0	0	0 (ND)
439C	63	1.5	0/34 (−)	100	20	0 (ND)

Information includes the age of the patient, the size of the tumor (mm), the expression percentage of estrogen receptor (ER), progesterone receptor (PR) and HER2, and the metastasis to the lymph node.

Lymph node status is indicated as (+) or (−); HER2 expression levels are grouped into four categories according to the pathological data: 3, high or overexpression (H) of HER2; 1–2, medium (M) expression; 1, low (L) expression, and 0 indicates that the expression was not detectable (ND).

In contrast, there was no strong association between the prevalence of ALDH^+^ cells and ER expression (Table 4, Figure 5B), which differed from other reports [34]. Among the specimens, we found that specimen #362C was the only one where high ALDH activity was associated with ER negative status (Table 4, Figure 5B). Furthermore, this specimen, and specimens #394C and #405C, were the only three with HER2 over-expression (Table 4). The high prevalence of ALDH^+^ cells in these three HER2-overexpression specimens provides a possibility that HER2 overexpression may drive tumorigenesis as well as ALDH expression as proposed [31]. However, such correlation was not in complete concordance since some specimens with a high prevalence of ALDH^+^ cells expressed low level of HER2 (Figure 5B).

In addition, we also observed a possible association between lymph node status and the prevalence of multiple markers. Specimens with a prevalence of CD133^+^ and CXCR4^+^ cells (#337C, #343C, #366C, #382C, #431C) or with a high prevalence of PROCR^+^ cells (#303C, #366C, #368C, #382C, #394C, #405C, #431C) had lymph node positive status (Table 4, Figure 5D, 5E, 5F). Thus, the association between each marker and ER, PR or HER2 or metastasis status in those clinical specimens implicated the existence of different breast cancer stem cells in patients reflecting with distinct clinical manifestation.

## Discussion

There is increasing evidence pointing to the existence of breast cancer stem cells and their central role in tumorigenesis. Two of the intriguing questions that need to be addressed are whether there is a uniform population or heterogeneous populations of breast cancer stem cells in one single tumor and whether these stem cell populations differ among different tumor types. We detected widely varying patterns of marker expression in different tumor specimens. Our data were consistent with some previously observed trends (negative correlation between CD44^+^/CD24^−/low^ and HER2) but differed from previous data in other regards (relationship of ALDH status and HER2 or ER). The widely varying marker expression in the tumor specimens strongly suggested the existence of more types of breast cancer stem cells than have been previously described. Experiments where we performed functional assays of tumorigenesis and *in vivo* tumor forming ability on marker expressing populations isolated from breast cancer cell lines also strongly supported this hypothesis. In particular, subpopulations of cells expressing markers PROCR and ESA were highly tumorigenic in both *in vitro* and *in vivo* assays. Thus PROCR and ESA expression defines as yet uncharacterized types of breast cancer stem cells and our data from both cell lines and primary specimens also suggest the existence of still more types of highly tumorigenic cells. These observations bring up two central questions. The first is whether the generally recognized markers that have been emphasized in previous studies of breast cancer stem cells actually identify the most highly tumorigenic cells. The second is whether there are different lineages of breast cancer stem cells which lead to different types of breast cancers and whether these different lineages may sometimes even coexist within the same tumor.

Most current studies have emphasized the identification of cancer stem cell subpopulations from breast tumors using CD44 and CD24. It has been reported that CD44^+^/CD24^−/low^ cells were more common in basal-like tumors and strongly associated with BRCA1 hereditary breast cancer; however, not every basal breast tumor contains CD44^+^/CD24^−/low^ cells [38]. Other recent studies have also shown that the presence of CD44^+^/CD24^−/low^ cells in breast tumors did not correlate with clinical outcome including tumor size, lymph node status or S-phase fraction [22], [39]. More recent data suggests that the newly identified marker ALDH could be more effective in identifying the most tumorigenic breast cancer stem cells [21]. However, only 30% of tumors contained ALDH^+^ cells. Although the ALDH phenotype correlates with clinical outcome such as tumor grade, no association with a particular molecular subtype of breast cancer was observed [21]. These findings raise the possibility that there might be cancer stem cells other than CD44^+^/CD24^−/low^ or ALDH^+^ that drive breast tumorigenesis. It has been reported that use of the surface marker CD133 can isolate a group of breast cancer stem cells that does not overlap with CD44^+^/CD24^−^ cells [23], which is consistent with our data that CD44^+^/CD24^−^ cells may not have the highest tumorigenic ability. In HCC1937, cells that lacked CD44^+^/CD24^−^ marker expression were capable of forming more colonies on soft agar than the CD44^+^/CD24^−^ cells (Figure 1B). These findings further suggest that CD44^+^/CD24^−^ can only enrich a subtype of breast cancer stem cell populations. On the other hand, we observed that cells with positive ALDH were not consistently correlated with their tumorigenicity. Although the ALDH^+^ ZR-75 cells exhibited higher tumorigenicity than bulk cells (Figure 1A), the ALDH marker failed to enrich higher tumorigenic cancer stem cells from the SKBR3 (Figure 1C) and HCC1937 cell lines (Figure S2, Method S1). These observations further demonstrated that CD44^+^/CD24^−/low^ and ALDH cannot serve as universal markers for cancer stem cell identification and isolation. Apparently, additional marker-identification study is needed to further define new cancer stem cell populations.

Different human breast cancer cell lines are known to have different abilities to form tumors *in vivo*. It is possible that this difference in tumorigenicity is due to the presence of different types of cancer stem cells. Similar to primary tumor specimens, different breast cancer cell lines had different cancer stem cell marker profiles as demonstrated in this study (Table 1). For each cell line, the subpopulations expressing cancer stem cell markers exhibited tumorigenic ability different from that of cells without marker expression. Using PROCR and ESA markers, we were able to isolate a subset of highly tumorigenic cells from the MDA-MB-231 cell line (the prevalence of CD44^+^/CD24^−/low^ in the bulk cell population was more than 90%) (Figure 1D and Table 2). Although it has been reported that CD44^+^ and PROCR^+^ cells were similar to each other and were enriched for genes involved in cell motility, chemotaxis, hemostasis, and angiogenesis as well as stem cell-specific genes [22], our data suggested that PROCR^+^/ESA^+^ allowed further enrichment of highly tumorigenic cancer stem cells from the CD44^+^/CD24^−/low^ breast cancer cells. In addition, our preliminary proteome analysis suggests that PROCR^+^/ESA^+^ cells exhibit a cancer stem cell molecular signature and that PROCR^+^/ESA^+^ and PROCR^−^/ESA^−^ MDA-MB-231 cells differ in expression of a number of proteins (WWHV et al unpublished data). For example, PROCR^+^/ESA^+^ cells showed a higher expression of casein kinase 2 which is involved in the Wnt signaling pathway [40] known to be highly activated in breast cancer stem cells. Such additional characterization of the tumorigenic cell populations has potential to clearly define the molecular signature of the small populations with highly tumorigenic activity.

It was a significant challenge to conduct experiments using primary tumor cells due to limited materials. To overcome this difficulty, *in vitro* culture and in animal xenograft tumor transplantations are required to expand cancer cells for assessing functional stem cell properties in primary tumors, albeit, the prevalence of marker expressing cells could potentially change under such selection. Overall, we processed 131 primary specimens through the course of this study; however, most of the tumors were too small for the marker expression profiling. We have amplified the tumor cells by inoculating them in NOD/SCID mice. However, only seven transplants with tumor outgrowth were observed, and only one of the seven was large enough for sorting by stem cell markers. Interestingly, selection with CD44^+^/CD24^−/low^ from this transplant enriched a subpopulation with higher soft agar colony forming ability (Figure S4, Method S1). Continued parallel analysis of primary tumor specimens will reveal which of these stem cell populations are of greatest importance in clinical outcome. Similar to the recent novel therapeutic strategies specifically targeting different cancer stem cells in different leukemias [41], further characterization of these breast cancer stem cells and the signaling pathways underlying their phenotypes will allow us to design tailored therapy to treat different types of breast cancers.

## Materials and Methods

### Human Breast Cancer Tissue Dissociation and Cell Preparation

#### Ethics statement

Human samples were collected after obtaining informed written consent from all participants. The samples were encoded to protect patient confidentiality and used under protocols approved by the Institutional Review Board of Human Subjects Research Ethics Committee of Academia Sinica (AS-IRB02-98042) and National Taiwan University, Taipei, Taiwan (#200902001R).

Human breast cancer tissue obtained from National Taiwan University Hospital was dissociated enzymatically and mechanically in a manner similar to previous reports but with modification [42]. In brief, tissue was minced into 2–3 mm^3^ pieces with sterile scalpels and enzymetically dissociated for 15–16 hours at 37°C in DMEM (Gibco/Invitrogen) supplemented with 150 units/mL collagenase (Sigma), 50 units/mL hyaluronidase and antibiotics/antimycotics. After centrifugation at 300 ×g for 5 min, red blood cells were removed. A single cell suspension was obtained by mechanical disaggregation in 0.25% trypsin for 5 min, followed by digestion in 200 µg/mL DNase1 for 1 min, and passage through a 40-µm cell strainer. Single cells were then transferred into mammary epithelium basal medium (MEBM) (Cambrex) supplemented with B27 (Invitrogen), 20 ng/mL EGF, 10 ng/mL FGF (Sigma), 4 µg/mL insulin (Invitrogen) and antibiotics/antimycotics. Hematopoietic and endothelial cells were removed using Dynabeads coated with antibodies against CD45, CD14, CD15, CD19, and CD31 (Invitrogen) following the manufacturers instructions. Cells were then subjected to stem cell marker profiling analysis.

### Cell Culture

Human breast cancer cell lines MCF-7, MDA-MB-231, MDA-MB-361, MDA-MB-468, T47D, ZR-75, SK-BR-3 and HCC-1937 were obtained from the American Type Culture Collection (ATCC) and routinely maintained in DMEM supplemented with 10% FBS and antibiotics/antimycotics in a humidified 37°C incubator supplemented with 5% CO_2_.

### Stem Cell Marker Profiling: Cell Labeling, Aldefluor Assay and Flow Cytometry

Cell labeling was done by staining with antibodies in buffer composed of PBS supplemented with 0.1% sodium azide, 1% FBS and 2 mM EDTA. Cells were first suspended and blocked in ice cold staining buffer at a concentration of 2.5×10^6^ cells/mL for 10 min, and stained with antibodies (using antibody titration suggested by the supplier) for 30 min on ice in the dark. After centrifugation at 300 ×g for 5 min at 4°C, cells were then washed 1–2 times with cold staining buffer before being subjected to flow cytometry. Antibodies used in this study were mouse(m)-anti(α)-human(h)-CD44-allophycocyanin (APC), mαhCD24-phycoerythrin (PE), rat-αhPROCR-PE, mαhCXCR4-APC (BD Bioscience), mαhABCG2-APC (R&D), mαhESA-647 (eBioscience), and m-αhCD133-APC (Miltenyi Biotec J&H Technology). Proper isotype controls were used for each cell labeling experiment. Aldefluor assay was performed following the manufacturer instruction using an Aldefluor kit (Stemcell Technologies). The stem cell marker profiling analysis was performed using a BD FACS Canto II. Live cell sorting was done using a BD FACS Aria with 100 µm nozzle following the manufacturer instructions. Sorted cells were washed with DMEM/10%FBS/antibiotics/antimycotics three times before being cultured in MEBM/B27 media supplemented with 20 ng/mL EGF, 10 ng/mL FGF and 4 µg/mL insulin. Cells were allowed to recover in ultra low attachment surface plates overnight in a humidified 37°C incubator before further analyses. The percentage of cells in different marker populations was evaluated using BD FACSDiva software.

### Soft Agar Colony Formation Assay

Soft agar colony formation assay was performed by seeding cells in a layer of 0.35% agar DMEM/FBS over a layer of 0.5% agar/DMED/FBS. Additional MEBM/B27/EGF/FGF/insulin media was added every 5 days to continuously supply growth supplements to the cells. Cultures were maintained in a humidified 37°C incubator. On day 14 or day 21 after seeding, cells were fixed with pure ethanol containing 0.05% crystal violet and colony forming efficiency quantified by light microscopy.

### Mouse Tumorigenicity Assay

NOD/SCID mice were used to evaluate the stem cell properties of sorted cells expressing potential stem cell markers from the human breast cancer cell lines. Animal care and experiments were approved by the Institutional Animal Care and Utilization Committee of Academia Sinica (IACUC#080085). The animal model was adapted and modified from Kuperwasser et al [43]. NOD/SCID fat pads were injected with sorted cancer cells mixed with human breast cancer associated fibroblasts (CAF) (1∶1) and Matrigel (BD bioscience) (1∶1). Tumor volumes were evaluated every five days after initial detection. The tumor formation efficiency was determined on day 50 after cell injection.

### Cell Cycle Profiling

MDA-MB-231 cells were synchronized by culturing in serum starvation condition (DMEM supplemented with 0.5% FBS) for 48 hours. After synchronization, the growth medium was replaced with DMEM supplemented with 10%FBS. Cells were then labeled with rat-αhPROCR-PE and mαhESA-647 antibodies and subjected to cell sorting to collect PROCR^+^/ESA^+^ and PROCR^−^/ESA^−^ subpopulations at 20, 24 and 48 h after released from starvation. Cells were washed twice with PBS and then fixed in ice-cold 70% ethanol overnight before stained with 10 µg/ml propidium iodide (PI). The PI stained cells were subjected to cell cycle profiling analysis on BD FACS Canto II reading at 488 nm.

### Real-Time RT-PCR

Quantitative real-time RT-PCR was performed using SYBR-Green master mix (Applied Biosystems) according to the manufacturer's instruction and analyzed on an ABI 7300 Real-Time PCR system. GAPDH mRNA was used as an internal control to normalize RNA inputs and expression levels were calculated according to the relative ΔC_t_ method. Primers used in this analysis were designed as previously described [19] and primer sequences are given in Table S1.

## Supporting Information

Figure S1 Estrogen receptor (ER) expression levels in bulk, ALDH^+^ and ALDH^−^ ZR-75 cells. A. ALDH^+^ and ALDH^−^ ZR-75 cells were collected for immunoblot analysis. Different expression levels of ERα were detected in the bulk, ALDH^+^ and ALDH^−^ cells. The expression levels of ERα in ALDH^−^ cells were 8-fold higher than the bulk cells, and 10-fold higher than the ALDH^+^ cells. B. Shown is the relative ERα expression levels in the bulk, ALDH^+^ and ALDH^−^ cells using the average from three independent immunoblotting assays (means± SE).
(0.03 MB PDF)

Figure S2 Soft agar colony forming efficiency of marker expressing or nonexpressing subpopulations from HCC1937 and ZR-75 cells. A. ALDH^+^ and CXCR4^+^ identified two distinct subpopulations of ZR-75 cells which did not overlap with each other. B. Both ALDH^+^ and CXCR4^+^ ZR-75 cells formed more colonies than the cells not expressing these markers. The ALDH^+^ cells were more tumorigenic than the CXCR4^+^ cells. Shown is the relative fold increase of the colony forming efficiency normalized to the colony forming efficiency of the bulk population (means ± SD). C. Selection of ALDH^+^ cells failed to enrich the tumorigenic potential of HCC1937 cells. Shown is the percentage of colony formation (means ± SD).
(0.02 MB PDF)

Figure S3 PROCR^+^/ESA^+^ MDA-MB-231 cells asymmetrically divide *in vivo*. A. PROCR^+^/ESA^+^ MDA-MB-231 cells with 93.8 percent purity were collected for *in vivo* inoculation in NOD/SCID mice. B. The marker profile of the cells derived from the tumor showed that the PROCR^+^/ESA^+^ cells retained at a small percentage (0.6%) and asymmetrically divided into PROCR^−^/ESA^−^ and PROCR^−^/ESA^+^ cells *in vivo*.
(0.02 MB PDF)

Figure S4 Stem cell marker profiling of primary breast cancer specimen #235C and the mammosphere and soft agar colony forming efficiency of CD44^+^/CD24^−/low^ cells compared to other cells from #235C. A. The prevalence of CD44^+^/CD24^−/low^, PROCR^+^, ESA^+^, ABCG2^+^, CXCR4^+^, CD133^+^ and ALDH^+^ cells were determined using flow cytometry. B. The mammosphere forming efficiency in CD44^+^/CD24^−/low^ cells was 4-fold higher than other cells. C. The soft agar colony forming efficiency in CD44^+^/CD24^−/low^ cells was 10-fold higher than other cells.
(0.02 MB PDF)

Table S1 Primer sequences.
(0.03 MB DOC)

Method S1 (0.04 MB DOC)
