# Supplementary figures and images for: Multiple Lineages of Human Breast Cancer Stem/Progenitor Cells Identified by Profiling with Stem Cell Markers
Source: PLoS One. 2009 Dec 21;4(12):e8377. doi: 10.1371/journal.pone.0008377 (PMC2793431; doi:10.1371/journal.pone.0008377)

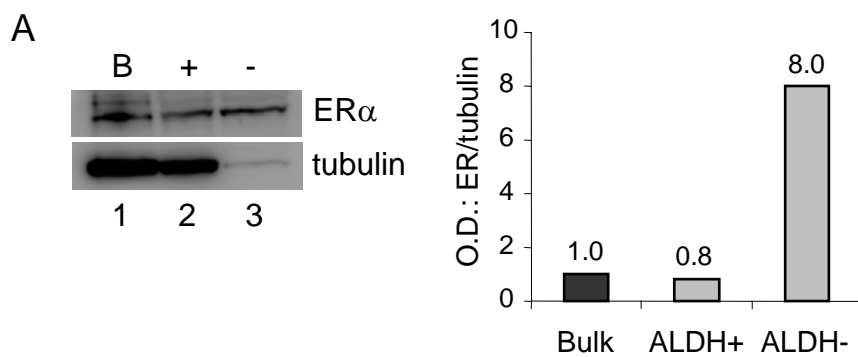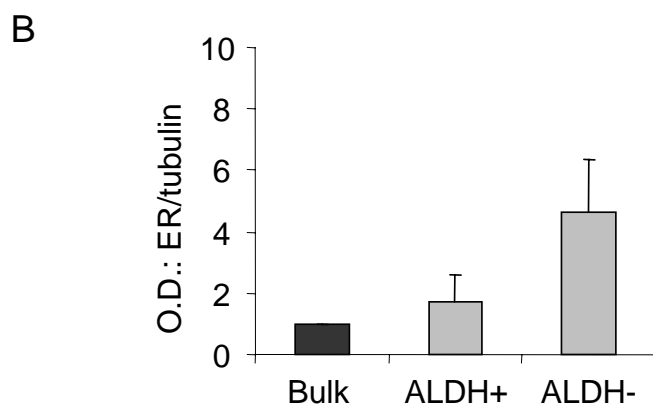

Supplement: Figure S1 — Estrogen receptor (ER) expression levels in bulk, ALDH+ and ALDH− ZR-75 cells. A. ALDH+ and ALDH− ZR-75 cells were collected for immunoblot analysis. Different expression levels of ERα were detected in the bulk, ALDH+ and ALDH− cells. The expression levels of ERα in ALDH− cells were 8-fold higher than the bulk cells, and 10-fold higher than the ALDH+ cells. B. Shown is the relative ERα expression levels in the bulk, ALDH+ and ALDH− cells using the average from three independent immunoblotting assays (means± SE). (0.03 MB PDF) [file pone.0008377.s001.pdf]
