# Supplementary material for: Multiple Lineages of Human Breast Cancer Stem/Progenitor Cells Identified by Profiling with Stem Cell Markers
Source: PLoS One. 2009 Dec 21;4(12):e8377. doi: 10.1371/journal.pone.0008377 (PMC2793431; doi:10.1371/journal.pone.0008377)

A ALDH⁺ and CXCR4⁺ ZR-75 cells did not overlap with each other.

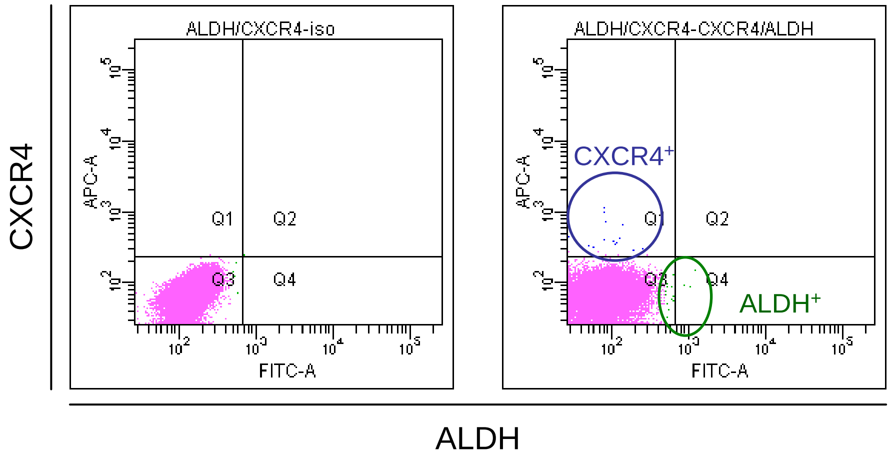

B ALDH⁺ ZR-75 cells exhibited higher tumorigenicity than CXCR4⁺ ZR-75 cells.

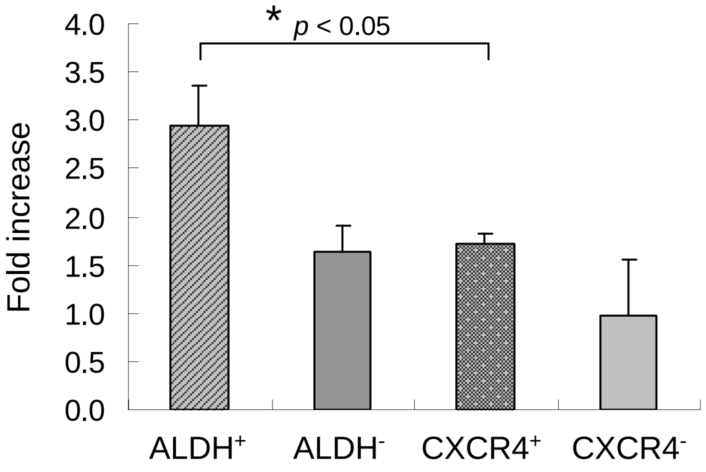

C Soft agar colony forming efficiency of ALDH⁺ and ALDH⁻ HCC1937 cells

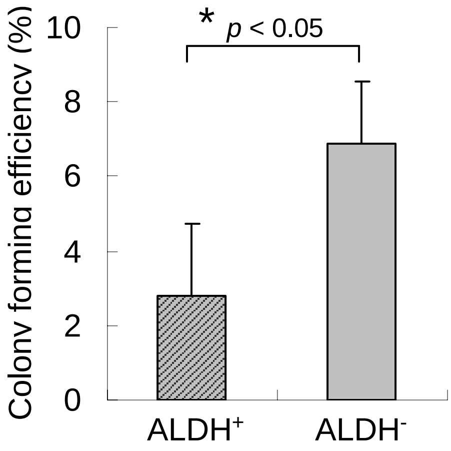

Supplement: Figure S2 — Soft agar colony forming efficiency of marker expressing or nonexpressing subpopulations from HCC1937 and ZR-75 cells. A. ALDH+ and CXCR4+ identified two distinct subpopulations of ZR-75 cells which did not overlap with each other. B. Both ALDH+ and CXCR4+ ZR-75 cells formed more colonies than the cells not expressing these markers. The ALDH+ cells were more tumorigenic than the CXCR4+ cells. Shown is the relative fold increase of the colony forming efficiency normalized to the colony forming efficiency of the bulk population (means ± SD). C. Selection of ALDH+ cells failed to enrich the tumorigenic potential of HCC1937 cells. Shown is the percentage of colony formation (means ± SD). (0.02 MB PDF) [file pone.0008377.s002.pdf]
