# Supplementary material for: Multiple Lineages of Human Breast Cancer Stem/Progenitor Cells Identified by Profiling with Stem Cell Markers
Source: PLoS One. 2009 Dec 21;4(12):e8377. doi: 10.1371/journal.pone.0008377 (PMC2793431; doi:10.1371/journal.pone.0008377)

A Sorted PROCR$^+$/ESA$^+$ MDA-MB-231 cells before injection into fat pad of NOD/SCID mice

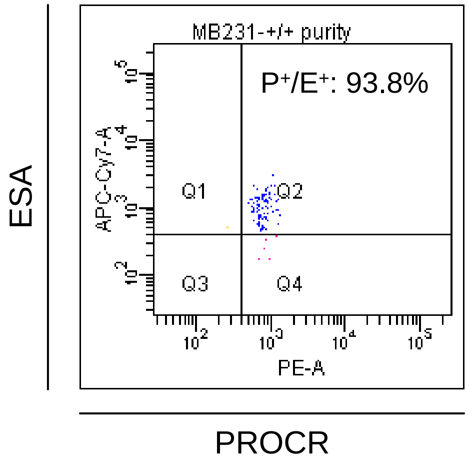

B PROCR/ESA expression profile in tumor derived from PROCR$^+$/ESA$^+$ MDA-MB-231 cells

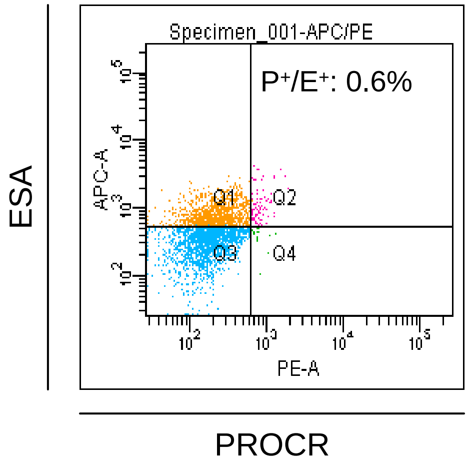

Supplement: Figure S3 — PROCR+/ESA+ MDA-MB-231 cells asymmetrically divide in vivo. A. PROCR+/ESA+ MDA-MB-231 cells with 93.8 percent purity were collected for in vivo inoculation in NOD/SCID mice. B. The marker profile of the cells derived from the tumor showed that the PROCR+/ESA+ cells retained at a small percentage (0.6%) and asymmetrically divided into PROCR−/ESA− and PROCR−/ESA+ cells in vivo. (0.02 MB PDF) [file pone.0008377.s003.pdf]
