# Supplementary material for: Multiple Lineages of Human Breast Cancer Stem/Progenitor Cells Identified by Profiling with Stem Cell Markers
Source: PLoS One. 2009 Dec 21;4(12):e8377. doi: 10.1371/journal.pone.0008377 (PMC2793431; doi:10.1371/journal.pone.0008377)

A The prevalence of solid cancer stem cell markers in a human breast cancer specimen #235C.

**Marker**	**Prevalence (%)**
$CD44^{+}/CD24^{-/low}$	0.5
$PROCR^{+}$	0
ESA^{+}	75.3
$ABCG2^{+}$	0.1
$CXCR4^{+}$	0.1
$CD133^{+}$	0
$ALDH^{+}$	0

B $CD44^{+}/CD24^{-/low}$ cells from #235C exhibited higher mammosphere forming ability than other cells

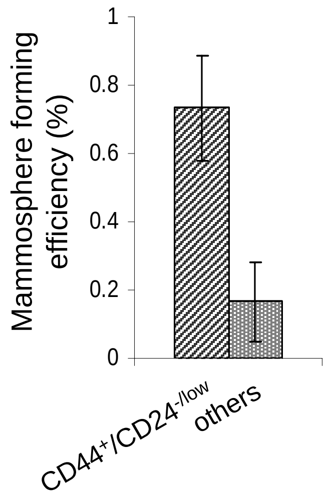

C $CD44^{+}/CD24^{-/low}$ cells from #235C exhibited higher colony forming ability than other cells

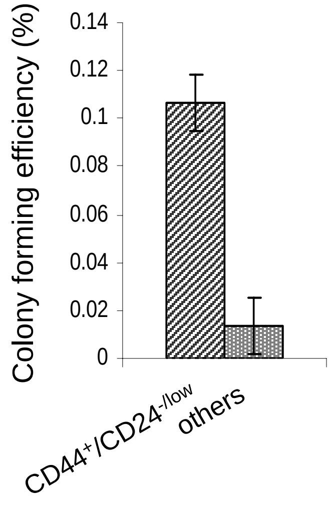

Supplement: Figure S4 — Stem cell marker profiling of primary breast cancer specimen #235C and the mammosphere and soft agar colony forming efficiency of CD44+/CD24−/low cells compared to other cells from #235C. A. The prevalence of CD44+/CD24−/low, PROCR+, ESA+, ABCG2+, CXCR4+, CD133+ and ALDH+ cells were determined using flow cytometry. B. The mammosphere forming efficiency in CD44+/CD24−/low cells was 4-fold higher than other cells. C. The soft agar colony forming efficiency in CD44+/CD24−/low cells was 10-fold higher than other cells. (0.02 MB PDF) [file pone.0008377.s004.pdf]
