# Supplementary material for: Multiple Lineages of Human Breast Cancer Stem/Progenitor Cells Identified by Profiling with Stem Cell Markers
Source: PLoS One. 2009 Dec 21;4(12):e8377. doi: 10.1371/journal.pone.0008377 (PMC2793431; doi:10.1371/journal.pone.0008377)
Supplement: Table S1 — Primer sequences. (0.03 MB DOC) [file pone.0008377.s005.doc]

Table S1. Primer sequences

| **Gene** | **Primer Sequence** |
| --- | --- |
| Numb | Forward 5’-GCTGCCTCTCCAGGTCTCTTC-3’  Reverse 5’-CGCTCTTAGACACCTCTTCTAACCA-3’ |
| vimentin | Forward 5’- GAGAACTTTGCCGTTGAAGC-3’  Reverse 5’- GCTTCCTGTAGGTGGCAATC-3’ |
| E-cadherin | Forward 5’- TGCCCAGAAAATGAAAAAGG-3’  Reverse 5’- GTGTATGTGGCAATGCGTTC-3’ |
| Slug | Forward 5’- GGGGAGAAGCCTTTTTCTTG-3’  Reverse 5’- TCCTCATGTTTGTGCAGGAG-3’ |
| FOXC2 | Forward 5’- GCCTAAGGACCTGGTGAAGC-3’  Reverse 5’- TTGACGAAGCACTCGTTGAG-3’ |
| GAPDH | Forward 5’- ACCCAGAAGACTGTGGATGG-3’  Reverse 5’- TCTAGACGGCAGGTCAGGTC-3’ |
