# Supplementary material for: Multiple Lineages of Human Breast Cancer Stem/Progenitor Cells Identified by Profiling with Stem Cell Markers
Source: PLoS One. 2009 Dec 21;4(12):e8377. doi: 10.1371/journal.pone.0008377 (PMC2793431; doi:10.1371/journal.pone.0008377)
Supplement: Method S1 — (0.04 MB DOC) [file pone.0008377.s006.doc]

**Hwang-Verslues et al, 2009**

**Supporting Information**

**Methods and Materials**

**Human breast cancer tissue dissociation and cell preparation**

All human samples were encoded to protect patient confidentiality and used under protocols approved by the Institutional Review Board of Human Subjects Research Ethics Committee of Academia Sinica (AS-IRB02-98042) and National Taiwan University, Taipei, Taiwan (#200902001R). Human breast cancer tissue obtained from NTU Hospital was dissociated enzymatically and mechanically in a manner similar to previous reports but with modification [42]. A single cell suspension was obtained and the hematopoietic and endothelial cells were removed as described in the main text. NOD/SCID mice were used to inoculate and amplify cancer cells by injecting NOD/SCID fat pads with cancer cells mixed with human breast cancer associated fibroblasts (CAF) (1:1) and Matrigel (BD bioscience) (1:1). When tumor volumes reached >1 cm in diameter, the tumors were collected. Cells were then subjected to stem cell marker profiling analysis. Cells expressing stem cell markers were isolated for mammosphere and soft agar colony formation assays.

**Mammosphere assay**

One thousand cells were cultured in MEBM/B27 media supplemented with 20 ng/mL EGF, 10 ng/mL FGF and 4 μg/mL insulin in a well of ultra low attachment surface 6-well plates in a humidified 37°C incubator. The supplements were added every 3-5 days and the numbers of mammospheres were counted on day 14 after seeding.

**Cell culture**

Human breast cancer cell lines MDA-MB-231, HCC1937 and ZR-75 were obtained from ATCC and routinely maintained in DMEM supplemented with 10% FBS and antibiotics/antimycotics in a humidified 37°C incubator supplemented with 5% CO_2_.

**Cell labeling and flow cytometry**

Cell labeling was done by staining with antibodies in buffer composed of PBS supplemented with 0.1% sodium azide, 1% FBS and 1-2mM EDTA. Cells were first suspended and blocked in ice cold staining buffer at a concentration of 2.5×10^6^ cells/mL for 10 min, and stained with antibodies (using antibody titration suggested by the supplier) for 30 min on ice in the dark. After centrifugation at 300 ×g for 5 min at 4°C, cells were then washed 1-2 times with cold staining buffer before being subjected to flow cytometry. Antibodies used in these supplemental experiments were mαhCXCR4-APC, rat-αhPROCR-PE (BD Bioscience), and mαhESA-647 (eBioscience). Proper isotype controls were used for each cell labeling experiment. Aldefluor assay was performed following the manufacturer instruction using an Aldefluor kit (Stemcell Technologies). Live cell sorting was done using a BD FACS Aria with 100 μm nozzle following the manufacturer’s instructions. Sorted cells were washed with DMEM/10%FBS/antibiotics/antimycotics three times before being cultured in MEBM/B27 media supplemented with 20 ng/mL EGF, 10 ng/mL FGF and 4 μg/mL insulin. Cells were allowed to recover in ultra low attachment surface plates overnight in a humidified 37°C incubator before further analyses. Quantification of the prevalence of the stem cell surface markers was performed using a BD FACS Canto II and analyzed using BD FACSDiva software.

**Immunoblot Analysis**

Immunoblot (IB) analysis was performed after 8% SDS-PAGE with overnight incubation of a 1:1000 dilution of anti-ERα antibody (MC-20, Santa Cruz Biotechnology, Santa Cruz, CA) followed by a 1:5000 dilution of horseradish peroxidase (HRP)-conjugated donkey anti-rabbit (DαR-HRP) antibody (Amersham, GEHealthcare). Signals were detected using Immobilon Western Chemiluminescent HRP Sabstrate (Millipore). Protein concentration was determined by the Bradford assay (Bio-Rad, Hercules, CA) prior to loading and verified by tubulin level using a 1:5000 dilution of anti-tubulin antibody (Abcam). The optical density was determined using NIH ImageJ program.

**Mouse tumorigenicity assay**

NOD/SCID mice were used to inoculate the tumors derived from the PROCR^+^/ESA^+^ MDA-MB-231 cells. Animal care was in accord with Academia Sinica Genomics Research Center guidelines (IACUC#080085). NOD/SCID fat pads were injected with sorted cancer cells mixed with human breast cancer associated fibroblasts (CAF) (1:1) and Matrigel (BD bioscience) (1:1). The bulk cells were isolated from the tumors, the mouse linage was excluded using FITC conjugated-antibody against mouse MHC Class I (eBioscience) and the cancer cells were evaluated for the prevalence of PROCR and ESA positive cells when the tumors reached about 1 cm in diameter.

**Soft agar colony formation assay**

Soft agar colony formation assay was performed by seeding cells in a layer of 0.35% agar DMEM/FBS over a layer of 0.5% agar/DMED/FBS. Additional MEBM/B27/EGF/FGF/insulin media was added every 5 days to continuously supply growth supplements to the cells. Cultures were maintained in a humidified 37°C incubator. On day 14 or day 21 after seeding, cells were fixed with pure ethanol containing 0.05% crystal violet and colony forming efficiency quantified by light microscopy.
